# Supplementary material for: Association of Dietary Patterns with Cardiovascular and Kidney Phenotypes in an Uruguayan Population Cohort
Source: Nutrients. 2021 Jun 27;13(7):2213. doi: 10.3390/nu13072213 (PMC8308311; doi:10.3390/nu13072213)
Supplement: Supplementary file 1 [file nutrients-13-02213-s001.zip › nutrients-1233218-supplementary.pdf]

SUPPLEMENTARY INFORMATION

Association of Dietary Patterns with Cardiovascular and Kidney Phenotypes in a Uruguayan Population Cohort

Paula Moliterno [1], Carmen Marino Donangelo [2], Luciana Borgarello [3], Matías Pécora [4], Alicia Olascoaga [3], Oscar Noboa [5] and José Boggia [5,*]

[1] Departamento de Nutrición Clínica. Escuela de Nutrición. Universidad de la República, Uruguay; pmoliterno@nutricion.edu.uy
[2] Escuela de Nutrición. Universidad de la República, Uruguay; cmarino@nutricion.edu.uy
[3] Laboratorio de Patología Clínica, Universidad de la República, Uruguay; luborga@hotmail.com (L.B.); aolascoa@gmail.com (A.O.)
[4] Departamento de Fisiopatología, Universidad de la República, Uruguay; pecora.matias@gmail.com
[5] Centro de Nefrología, Universidad de la República, Uruguay; onoboa@gmail.com (O.N.); jboggia@hc.edu.uy (J.B.)
* Correspondence: jboggia@hc.edu.uy

Number of Tables 7

Table S1. Characteristics of participants according to age categories [ǂ].

**Variable**	**Age ≤ 54 years (n=138)**	**Age > 54 years (n=156)**	***p-Value****
Female	94 (68.1)	105 (67.3)	0.88
Education ≤ 9 years	74 (53.6)	115 (73.7)	0.0003
Low physical activity	101 (73.2)	121 (77.6)	0.38
Current smokers	31 (24.4)	24 (16.2)	0.090
Alcohol use	48 (37.8)	49 (33.1)	0.42
Hypertension	20 (14.5)	87 (55.8)	<0.0001
On antihypertensive drugs †	12 (60.0)	70 (80.5)	0.076
Uncontrolled Hypertension -OBP	13 (9.4)	47 (30.1)	<0.0001
Uncontrolled Hypertension -ABPM	41 (36.6)	16 (19.8)	0.011
Diabetes	2 (1.5)	28 (18.6)	<0.0001
Obesity	54 (39.1)	62 (39.7)	0.91
Vitamin D deficiency	13 (9.4)	10 (6.4)	0.34
Chronic kidney disease	3 (3.4)	8 (11.9)	0.057
Nephrolithiasis	11 (8.0)	26 (16.7)	0.025
Fasting glucose (mg/dl)	89.6±10.9	104.2±33.2	<0.0001
Total cholesterol (mg/dl)	195.8±34.7	214.6±46.6	<0.0001
Triglyceride (mg/dl)	124.4±73.7	146.3±80.3	0.016
LDLc (mg/dl)	117.1±31.3	132.7±37.4	0.0001
HDLc (mg/dl)	52.6±13.4	51.8±14.5	0.62
Non-HDL cholesterol	141.9±35.2	162.9±43.9	<0.0001
TG/HDLc ratio	2.67±2.19	3.23±2.41	0.040
Waist/height ratio	0.57±0.11	0.60±0.077	0.0097
BMI (kg/m^2)	29.5±7.6	29.5±5.1	0.98
HOMA-r	3.06±2.43	3.48±3.20	0.20
Neutrophils/Lymphocytes ratio	1.74±0.86	1.80±0.77	0.55
Serum 25(OH)D (ng/ml)	23.8±9.9	25.0±9.6	0.26
Systolic blood pressure (mm Hg)	118.0±13.6	130.5±18.6	<0.0001
Diastolic blood pressure (mm Hg)	77.4±8.9	81.5±10.3	0.0003
Pulse wave velocity (m/s)	7.1±1.5	10.0±3.1	<0.0001
Serum creatinine	0.74±0.15	0.81±0.24	0.0015
Proteinuria (g/24-h)	0.14±0.085	0.14±0.14	0.54
Albumin/creatinine ratio (mg/g)	6.1±15.5	14.6±37.2	0.083
eGFR (mL/min/1.73 m^2)	108.5±15.8	83.8±17.0	<0.0001

[ǂ] Values are mean±SD for continuous variables and *n* (%) for categorical variables.
* *p*-Value for X^2 Test for categorical variables and T Student for continuous variables. †Of the hypertensive patients. OBP office blood pressure; ABPM ambulatory blood pressure monitoring (*n* =193); LDLc low-density lipoproteins cholesterol; HDLc high-density lipoproteins cholesterol; TG/HDLc triglycerides/high-density lipoproteins cholesterol ratio; BMI body mass index; HOMA-r Homeostasis Model Assessment of Insulin Resistance; 25(OH)D 25-hydroxy-vitamin-D; eGFR Estimated glomerular filtration rate, derived from the Chronic Kidney Disease (CKD) Epidemiology Collaboration equation. Low physical activity was defined as energy expenditure <600mets/min per week. Current smokers (yes/no), alcohol use was considered if reported drinking alcohol at least once a week (yes/no). Hypertension was defined as blood pressure of at least 140 mm Hg systolic or 90 mm Hg diastolic or use of antihypertensive drugs. Uncontrolled hypertension was defined by office blood pressure of at least 140 mm Hg systolic or 90 mm Hg diastolic, and by ABPM of at least 130 mm Hg systolic or 80 mm Hg diastolic. Diabetes was defined as self-reported diagnosis, a fasting plasma glucose of 126 mg/dl or higher, or use of antidiabetic drugs. Obesity was defined as BMI ≥30 kg/m^2. Vitamin D deficiency was considered serum 25(OH)D <12 ng/ml. CKD was defined as an eGFR < 60 mL/min/1.73m^2 or an albumin/creatinine ratio > 30 mg/g, based on a single determination (*n* =155). Nephrolithiasis was defined according to report (yes/no). Pulse wave velocity was the distance in meters divided by the transit time in seconds (*n* =174).

Table S2. Characteristics of participants according to sex ǂ.

**Variable**	**Women (n=199)**	**Men (n=95)**	***p-Value****
Age (years)	53.4±16.4	51.9±17.5	0.47
Education ≤ 9 years	127 (65.3)	62 (63.8)	0.81
Low physical activity	161 (80.9)	61 (64.2)	0.002
Current smokers	43 (23.4)	12 (13.2)	0.047
Alcohol use	50 (27.2)	47 (51.7)	<0.0001
Hypertension	75 (37.7)	32 (33.7)	0.50
On antihypertensive drugs †	58 (77.3)	24 (75.0)	0.79
Uncontrolled Hypertension -OBP	42 (21.1)	18 (19.0)	0.67
Uncontrolled Hypertension -ABPM	32 (24.1)	25 (41.7)	0.013
Diabetes	24 (12.1)	7 (7.4)	0.22
Obesity	80 (40.2)	36 (37.9)	0.71
Vitamin D deficiency	20 (10.1)	3 (3.2)	0.04
Chronic kidney disease	3 (5.4)	8 (8.1)	0.75
Nephrolithiasis	18 (9.1)	19 (20.0)	0.0081
Fasting glucose (mg/dl)	97.9±30.3	96.0±14.6	0.46
Total cholesterol (mg/dl)	207.9±41.0	201.3±45.1	0.21
Triglyceride (mg/dl)	133.0±78.6	142.3±76.5	0.34
LDLc (mg/dl)	125.4±34.1	125.3±38.4	0.97
HDLc (mg/dl)	54.7±14.6	46.8±11.0	<0.0001
Non-HDL cholesterol	152.9±39.7	153.3±44.7	0.95
TG/HDLc ratio	2.75±2.19	3.42±2.52	0.022
Waist/height ratio	0.58±0.095	0.60±0.087	0.34
BMI (kg/m^2)	29.4±6.5	29.8±6.1	0.57
HOMA-r	3.20±3.13	3.44±2.23	0.46
Neutrophils/Lymphocytes ratio	1.76±0.72	1.80±0.99	0.69
Serum 25(OH)D (ng/ml)	22.3±9.6	28.8±8.5	<0.0001
Systolic blood pressure (mm Hg)	124.7±18.8	124.5±14.8	0.89
Diastolic blood pressure (mm Hg)	79.2±10.3	80.5±8.9	0.27
Pulse wave velocity (m/s)	8.8±2.8	9.2±3.3	0.37
Serum creatinine	0.71±0.18	0.92±0.18	<0.0001
Proteinuria (g/24-h)	0.14±0.13	0.14±0.098	0.83
Albumin/creatinine ratio (mg/g)	9.2±21.8	10.6±35.2	0.79
eGFR (mL/min/1.73 m^2)	95.8±19.9	94.5±22.0	0.62

ǂ Values are mean±SD for continuous variables and *n* (%) for categorical variables.

* *p*-Value for X^2 Test for categorical variables and T Student for continuous variables. †Of the hypertensive patients. OBP office blood pressure; ABPM ambulatory blood pressure monitoring (*n* =193); LDLc low-density lipoproteins cholesterol; HDLc high-density lipoproteins cholesterol; TG/HDLc triglycerides/high-density lipoproteins cholesterol ratio; BMI body mass index; HOMA-r Homeostasis Model Assessment of Insulin Resistance; 25(OH)D 25-hydroxy-vitamin-D; eGFR Estimated glomerular filtration rate, derived from the Chronic Kidney Disease Epidemiology Collaboration equation. Low physical activity was defined as energy expenditure <600mets/min per week. Current smokers (yes/no), alcohol use was considered if reported drinking alcohol at least once a week (yes/no). Hypertension was defined as blood pressure of at least 140 mm Hg systolic or 90 mm Hg diastolic or use of antihypertensive drugs. Uncontrolled hypertension was defined by office blood pressure of at least 140 mm Hg systolic or 90 mm Hg diastolic; and by ABPM of at least 130 mm Hg systolic or 80 mm Hg diastolic. Diabetes was defined as self-reported diagnosis, a fasting plasma glucose of 126 mg/dl or higher, or use of antidiabetic drugs. Obesity was defined as BMI ≥30 kg/m^2. Vitamin D deficiency was considered serum 25(OH)D <12 ng/ml. CKD was defined as an eGFR < 60 mL/min/1.73m^2 or an albumin/creatinine ratio > 30 mg/g, based on a single determination (*n* =155). Nephrolithiasis was defined according to report (yes/no). Pulse wave velocity was the distance in meters divided by the transit time in seconds (*n* =174).

Table S3. Demographic and anthropometric characteristics, conventional risk factors and cardiovascular and renal phenotypes by tertiles (T) of dietary pattern and age categories in Uruguayan adults.

	Age ≤ 54 years N=138				Age > 54 years N=156			
	**T1**	**T2**	**T3**	*p**	**T1**	**T2**	**T3**	*p**
**Meat DP**								
n	26	48	64		71	51	34	
Female, *n* (%)	22 (84.6)	32 (66.7)	40 (62.5)	0.058	52 (73.2)	36 (70.6)	17 (50.0)	0.028
Education ≤ 9 years, *n* (%)	12 (46.2)	20 (41.7)	42 (65.6)	0.031	54 (77.1)	34 (66.7)	26 (76.5)	0.72
Low physical activity, *n* (%)	20 (76.9)	33 (68.8)	48 (75.0)	0.96	55 (77.5)	40 (78.4)	26 (76.5)	0.94
Current smokers, *n* (%)	1 (4.2)	11 (24.4)	19 (32.7)	0.0082	6 (8.7)	7 (14.9)	11 (34.4)	0.0018
Alcohol use, *n* (%)	6 (25.0)	14 (31.1)	28 (48.3)	0.027	22 (31.9)	12 (25.5)	15 (46.9)	0.24
Obesity, *n* (%)	7 (26.9)	21 (43.8)	26 (40.6)	0.34	32 (45.1)	19 (37.3)	11 (32.4)	0.19
Hypertension, *n* (%)	3 (11.5)	8 (16.7)	9 (14.1)	0.88	43 (60.6)	32 (62.8)	12 (35.3)	0.033
Uncontrolled HT by office measurement, *n* (%)	2 (7.7)	7 (14.6)	4 (6.3)	0.54	25 (35.2)	16 (31.4)	6 (17.7)	0.081
Uncontrolled HT by ABPM measurement, *n* (%)	3 (17.7)	4 (15.4)	9 (23.7)	0.51	18 (33.3)	12 (32.4)	11 (52.4)	0.19
Pulse wave velocity (m/s)ǂ	7.1±1.3	7.3±1.2	6.9±1.9	0.60	9.7±2.9	10.9±3.3	9.2±2.9	0.83
Arterial stiffness, *n* (%)	0 (0)	0 (0)	1 (4.6)	0.23	22 (40.0)	17 (53.1)	4 (18.2)	0.21
Diabetes, *n* (%)	0 (0)	1 (2.1)	1 (1.6)	0.67	15 (21.1)	10 (19.6)	4 (11.8)	0.28
CKD, *n* (%)	1 (3.9)	0 (0)	2 (3.1)	0.89	8 (11.3)	6 (11.8)	4 (12.1)	0.89
Nephrolithiasis, *n* (%)	0 (0)	5 (10.4)	6 (9.4)	0.22	13 (18.3)	7 (13.7)	6 (17.7)	0.82
**Prudent DP**								
n	54	44	40		43	54	59	
Female, *n* (%)	35 (64.8)	33 (75.0)	26 (65.0)	0.90	27 (62.8)	33 (61.1)	45 (76.3)	0.12
Education ≤ 9 years, *n* (%)	30 (55.6)	27 (61.4)	17 (42.5)	0.25	38 (88.4)	39 (72.2)	37 (63.8)	0.0063
Low physical activity, *n* (%)	42 (77.8)	31 (70.5)	28 (70.0)	0.38	33 (76.7)	44 (81.5)	44 (74.6)	0.74
Current smokers, *n* (%)	11 (20.8)	7 (19.4)	13 (34.2)	0.16	6 (14.0)	10 (19.6)	8 (14.8)	0.95
Alcohol use, *n* (%)	19 (35.9)	14 (38.9)	15 (39.5)	0.72	12 (27.9)	18 (35.3)	19 (35.2)	0.47

Obesity, *n* (%)	22 (40.7)	21 (47.7)	11 (27.5)	0.24	15 (34.9)	27 (50.0)	20 (33.9)	0.78
Hypertension, *n* (%)	6 (11.1)	8 (18.2)	6 (15.0)	0.55	22 (51.2)	33 (61.1)	32 (54.2)	0.83
Uncontrolled HT by office measurement, *n* (%)	2 (3.7)	6 (13.6)	5 (12.5)	0.12	13 (30.2)	16 (29.6)	18 (30.5)	0.97
Uncontrolled HT by ABPM measurement, *n* (%)	4 (12.9)	7 (26.9)	5 (20.8)	0.42	12 (46.2)	12 (29.3)	17 (37.8)	0.62
Pulse wave velocity (m/s)ǂ	6.9±1.5	7.4±1.6	7.0±1.3	0.90	8.8±3.4	11.1±3.2	9.7±2.6	0.52
Arterial stiffness, *n* (%)	0 (0)	1 (4.6)	0 (0)	0.92	7 (29.2)	21 (61.8)	15 (29.4)	0.51
Diabetes, *n* (%)	2 (3.7)	0 (0)	0 (0)	0.12	6 (14.0)	11 (20.4)	12 (20.3)	0.44
CKD, *n* (%)	1 (1.9)	1 (2.3)	1 (2.5)	0.83	2 (4.8)	10 (18.5)	6 (10.2)	0.53
Nephrolithiasis, *n* (%)	5 (9.3)	3 (6.8)	3 (7.5)	0.73	9 (20.9)	9 (16.7)	8 (13.6)	0.33
**Cereal and Mate DP**								
n	51	41	46		47	56	53	
Female, *n* (%)	27 (52.9)	34 (82.9)	33 (71.7)	0.040	36 (76.6)	43 (76.8)	26 (49.1)	0.0027
Education ≤ 9 years, *n* (%)	22 (43.1)	19 (46.3)	33 (71.7)	0.0053	30 (65.2)	47 (83.9)	37 (69.8)	0.67
Low physical activity, *n* (%)	30 (58.8)	33 (80.5)	38 (82.6)	0.0075	34 (72.3)	46 (82.1)	41 (77.4)	0.57
Current smokers, *n* (%)	9 (18.4)	9 (23.1)	13 (33.3)	0.11	2 (4.7)	10 (18.9)	12 (23.1)	0.017
Alcohol use, *n* (%)	17 (34.0)	12 (31.6)	19 (48.7)	0.18	12 (27.9)	13 (24.5)	24 (46.2)	0.049
Obesity, *n* (%)	20 (39.2)	15 (36.6)	19 (41.3)	0.84	19 (40.4)	25 (44.6)	18 (34.0)	0.49
Hypertension, *n* (%)	9 (17.6)	5 (12.2)	6 (13.0)	0.51	23 (48.9)	35 (62.5)	29 (54.7)	0.59
Uncontrolled HT by office measurement, *n* (%)	7 (13.7)	2 (4.9)	4 (8.7)	0.38	11 (23.4)	20 (35.7)	16 (30.2)	0.49
Uncontrolled HT by ABPM measurement, *n* (%)	3 (12.0)	8 (30.8)	5 (16.7)	0.73	12 (37.5)	12 (30)	17 (42.5)	0.61
Pulse wave velocity (m/s)ǂ	7.1±1.4	7.0±1.7	7.3±1.3	0.65	9.6±3.0	10.1±2.7	10.1±3.4	0.48
Arterial stiffness, *n* (%)	0 (0)	1 (4.6)	0 (0)	0.98	11 (36.7)	17 (46.0)	15 (35.7)	0.86
Diabetes, *n* (%)	0 (0)	0 (0)	2 (4.4)	0.078	9 (19.2)	11 (19.6)	9 (17.0)	0.77
CKD, *n* (%)	0 (0)	1 (2.4)	2 (4.4)	0.14	4 (8.7)	9 (16.1)	5 (9.4)	0.95
Nephrolithiasis, *n* (%)	5 (9.8)	4 (9.8)	2 (4.4)	0.33	8 (17.0)	10 (17.9)	8 (15.1)	0.79

**p*-Value for trend ǂMean values±SD. HT: hypertension; ABPM ambulatory blood pressure monitoring (*n*=193); CKD Chronic Kidney Disease.

Low physical activity was defined as energy expenditure < 600mets/min per week. Current smokers (yes/no). Alcohol use was considered if reported drinking alcohol at least once a week (yes/no). Obesity was defined as BMI ≥30 kg/m². Hypertension was defined as blood pressure (BP) of at least 140 mm Hg systolic or 90 mm Hg

diastolic or use of antihypertensive drugs. Uncontrolled hypertension was defined by office BP of at least 140 mm Hg systolic or 90 mm Hg diastolic. Uncontrolled hypertension by ABPM was defined as BP of at least 130 mm Hg systolic or 80 mm Hg diastolic. Pulse wave velocity was the distance in meters divided by the transit time in seconds (*n* =174). Arterial stiffness was defined as a pulse wave velocity > 10 m/s. Diabetes was defined as self-reported diagnosis, a fasting plasma glucose of 126 mg/dl or higher, or use of antidiabetic drugs. CKD was defined as an eGFR < 60 mL/min/1.73m^2 or an albumin/creatinine ratio > 30 mg/g, based on a single determination (*n* =155). Nephrolithiasis was defined according to report (yes/no).

Table S4. Median metabolic, anthropometric, cardiovascular and renal variables according to tertiles (T) of dietary pattern and age categories in Uruguayan adults.

	**Age ≤ 54 years N=138**				**Age > 54 years N=156**			
	**T1**	**T2**	**T3**	***p****	**T1**	**T2**	**T3**	***p****
**Meat DP**								
Metabolic characteristics								
Fasting glucose (mg/dl)	87.6	86.1	88.7		98.7	99.6	107.0	
Adjusted median difference	Ref	-0.27	-0.72	0.63	Ref	0.71	2.13	0.64
Total cholesterol (mg/dl)	202.2	200.6	196.1		214.8	211.4	198.6	
Adjusted median difference	Ref	-0.67	-1.77	0.72	Ref	-2.52	-7.53	0.24
Triglyceride (mg/dl)	115.4	119.8	145.6		154.0	145.0	159.7	
Adjusted median difference	Ref	8.43	22.19	0.036	Ref	1.97	5.88	0.60
LDLc (mg/dl)	121.8	122.5	116.9		134.1	131.3	127.4	
Adjusted median difference	Ref	-0.87	-2.29	0.60	Ref	-1.30	-3.89	0.44
HDLc (mg/dl)	53.2	55.1	47.7		47.1	48.5	44.4	
Adjusted median difference	Ref	-1.37	-3.61	0.047	Ref	-1.06	-3.16	0.087
Non-HDL colesterol (mg/dl)	147.1	145.2	146.3		167.5	162.2	156.9	
Adjusted median difference	Ref	0.19	0.51	0.92	Ref	-1.38	-4.13	0.50
TG/HDLc ratio	2.53	2.50	3.42		3.67	3.53	4.08	
Adjusted median difference	Ref	0.33	0.88	0.0038	Ref	0.11	0.31	0.33
HOMA-r	2.33	2.52	3.10		3.11	3.25	4.04	
Adjusted median difference	Ref	0.15	0.40	0.15	Ref	0.19	0.55	0.17
Neutrophils/Lymphocytes ratio	1.86	1.83	1.89		1.89	1.93	1.82	
Adjusted median difference	Ref	0.033	0.086	0.48	Ref	0.048	0.14	0.18
Serum 25(OH)D (ng/ml)	23.2	22.7	25.4		28.8	25.6	25.5	
Adjusted median difference	Ref	0.39	1.01	0.42	Ref	-0.98	-2.94	0.012
Anthropometric characteristics								
BMI (kg/m^2)	25.2	28.6	28.3		28.2	27.8	27.6	
Adjusted median difference	Ref	0.35	0.93	0.37	Ref	0.016	0.049	0.94
Waist/height ratio	0.56	0.57	0.57		0.60	0.60	0.59	
Adjusted median difference	Ref	0.0031	0.0083	0.31	Ref	-0.00093	-0.0028	0.63
Cardiovascular and renal characteristics								
Systolic office BP (mm Hg)	120.6	124.7	123.3		127.5	127.1	123.6	
Adjusted median difference	Ref	-0.68	-1.79	0.33	Ref	-0.74	-2.21	0.35
Diastolic office BP (mm Hg)	77.2	83.0	79.1		83.5	81.6	80.6	
Adjusted median difference	Ref	-0.30	-0.78	0.52	Ref	-0.42	-1.26	0.34
Systolic ABPM (mm Hg)	118.4	120.6	122.3		121.0	125.2	123.8	
Adjusted median difference	Ref	0.27	0.75	0.78	Ref	0.70	2.10	0.34
Diastolic ABPM (mm Hg)	75.3	76.3	76.6		76.5	77.5	79.9	
Adjusted median difference	Ref	0.042	0.12	0.96	Ref	0.74	2.20	0.19
Pulse wave velocity (m/s)	7.6	7.7	7.1		9.0	10.2	9.2	
Adjusted median difference	Ref	-0.26	-0.67	0.055	Ref	0.084	0.22	0.68
Serum creatinine (mg/dl)	0.76	0.76	0.78		0.87	0.85	0.88	
Adjusted median difference	Ref	0.0014	0.0036	0.84	Ref	-0.0087	-0.026	0.39
Proteinuria (g/24-h)	0.13	0.12	0.16		0.16	0.12	0.18	
Adjusted median difference	Ref	0.014	0.038	0.0070	Ref	-0.0022	-0.0066	0.81
Albumin/creatinine ratio (mg/g)	5.1	1.3	6.1		28.7	19.1	28.5	

Adjusted median difference	Ref	0.82	2.16	0.49	Ref	-0.89	-2.64	0.73
eGFR (mL/min/1.73 m[2])	108.7	109.1	110.4		82.9	83.8	85.4	
Adjusted median difference	Ref	0.96	2.52	0.25	Ref	1.31	3.92	0.099
**Prudent DP**								
Metabolic characteristics								
Fasting glucose (mg/dl)	86.2	85.3	90.5		98.0	95.5	110.9	
Adjusted median difference	Ref	0.96	2.77	0.084	Ref	2.63	6.19	0.23
Total cholesterol (mg/dl)	192.7	198.3	204.1		213.4	198.4	207.1	
Adjusted median difference	Ref	2.70	7.78	0.13	Ref	1.93	4.54	0.54
Triglyceride (mg/dl)	129.4	139.9	131.0		139.7	168.0	142.7	
Adjusted median difference	Ref	1.69	4.87	0.67	Ref	-3.22	-7.60	0.55
LDLc (mg/dl)	115.7	117.0	125.2		121.7	133.1	133.0	
Adjusted median difference	Ref	2.22	6.39	0.17	Ref	3.06	7.20	0.22
HDLc (mg/dl)	51.4	49.4	52.1		50.7	45.1	46.3	
Adjusted median difference	Ref	0.091	2.62	0.89	Ref	-0.59	-1.40	0.51
Non-HDL colesterol (mg/dl)	140.1	144.5	152.7		148.2	169.6	160.7	
Adjusted median difference	Ref	2.93	8.44	0.10	Ref	2.28	3.37	0.44
TG/HDLc ratio	2.87	3.25	2.89		3.25	4.14	3.60	
Adjusted median difference	Ref	0.025	0.073	0.83	Ref	-0.054	-0.13	0.74
HOMA-r	2.67	2.33	3.27		2.52	3.30	4.16	
Adjusted median difference	Ref	0.13	0.38	0.21	Ref	0.26	0.62	0.18
Neutrophils/Lymphocytes ratio	1.92	2.00	1.71		1.75	1.91	1.90	
Adjusted median difference	Ref	-0.034	-0.099	0.45	Ref	0.053	0.13	0.31
Serum 25(OH)D (ng/ml)	23.3	21.9	26.1		28.4	25.2	27.2	
Adjusted median difference	Ref	0.71	2.05	0.12	Ref	0.096	0.23	0.87
Anthropometric characteristics								
BMI (kg/m[2])	28.3	29.4	27.2		25.8	29.2	27.9	
Adjusted median difference	Ref	-0.16	-0.47	0.68	Ref	0.61	1.44	0.070
Waist/height ratio	0.57	0.58	0.56		0.60	0.60	0.60	
Adjusted median difference	Ref	-0.00058	-0.0017	0.85	Ref	-0.0039	-0.0093	0.16
Cardiovascular and renal characteristics								
Systolic office BP (mm Hg)	123.4	122.3	124.3		122.8	127.0	126.8	
Adjusted median difference	Ref	-0.053	-0.15	0.94	Ref	0.54	1.28	0.64
Diastolic office BP (mm Hg)	79.1	79.8	81.4		80.3	81.0	83.8	
Adjusted median difference	Ref	0.45	1.31	0.32	Ref	0.45	1.07	0.49
Systolic ABPM (mm Hg)	121.4	123.9	119.9		125.5	123.2	122.2	
Adjusted median difference	Ref	-0.32	-0.88	0.77	Ref	-0.84	-2.31	0.29
Diastolic ABPM (mm Hg)	75.5	79.0	75.8		78.3	77.3	78.1	
Adjusted median difference	Ref	0.21	0.59	0.80	Ref	-0.12	-0.33	0.84
Pulse wave velocity (m/s)	7.1	7.6	7.4		8.1	10.0	9.4	
Adjusted median difference	Ref	-0.013	-0.039	0.90	Ref	0.11	0.25	0.66
Serum creatinine (mg/dl)	0.74	0.78	0.80		0.85	0.89	0.85	
Adjusted median difference	Ref	0.020	0.057	0.0027	Ref	-0.0069	-0.016	0.64
Proteinuria (g/24-h)	0.15	0.14	0.14		0.15	0.17	0.13	
Adjusted median difference	Ref	-0.0030	-0.0087	0.54	Ref	-0.0066	-0.016	0.58
Albumin/creatinine ratio (mg/g)	4.60	4.60	4.07		20.4	22.8	33.3	
Adjusted median difference	Ref	-0.056	-0.16	0.96	Ref	1.79	4.28	0.71
eGFR (mL/min/1.73 m[2])	114.2	107.7	106.8		85.5	82.1	85.5	
Adjusted median difference	Ref	-2.39	-6.88	0.0031	Ref	0.70	1.64	0.55

**Cereal and Mate DP**								
Metabolic characteristics								
Fasting glucose (mg/dl)	88.2	86.1	87.7		98.5	103.2	102.5	
Adjusted median difference	Ref	-0.60	-1.65	0.37	Ref	1.08	2.50	0.66
Total cholesterol (mg/dl)	199.6	197.8	197.1		208.5	204.8	209.9	
Adjusted median difference	Ref	-1.60	-4.36	0.46	Ref	4.14	9.59	0.23
Triglyceride (mg/dl)	135.1	132.1	129.7		174.3	137.9	152.5	
Adjusted median difference	Ref	2.13	5.80	0.66	Ref	-0.32	-0.74	0.96
LDLc (mg/dl)	118.8	121.7	116.6		126.0	128.5	134.3	
Adjusted median difference	Ref	-2.91	-7.95	0.13	Ref	4.45	10.29	0.10
HDLc (mg/dl)	48.6	50.7	55.6		46.9	47.9	45.9	
Adjusted median difference	Ref	1.83	5.01	0.024	Ref	-0.27	-0.62	0.79
Non-HDL colesterol (mg/dl)	150.1	142.5	142.1		159.9	158.8	164.8	
Adjusted median difference	Ref	-2.95	-8.05	0.17	Ref	4.82	11.5	0.14
TG/HDLc ratio	3.16	3.00	2.68		4.22	3.39	3.78	
Adjusted median difference	Ref	-0.071	-0.19	0.61	Ref	-0.020	-0.047	0.91
HOMA-r	2.80	2.85	2.77		3.21	3.85	3.37	
Adjusted median difference	Ref	-0.055	-0.15	0.66	Ref	0.074	0.17	0.74
Neutrophils/Lymphocytes ratio	1.67	2.01	2.05		2.01	1.96	1.77	
Adjusted median difference	Ref	0.11	0.29	0.051	Ref	-0.027	-0.062	0.65
Serum 25(OH)D (ng/ml)	24.2	23.7	24.0		25.6	29.1	25.5	
Adjusted median difference	Ref	0.31	0.84	0.60	Ref	0.19	0.45	0.76
Anthropometric characteristics								
BMI (kg/m^2)	28.3	28.3	27.9		27.6	27.3	28.4	
Adjusted median difference	Ref	-0.093	-0.25	0.84	Ref	0.14	0.33	0.71
Waist/height ratio	0.57	0.55	0.57		0.60	0.60	0.60	
Adjusted median difference	Ref	0.0021	0.0058	0.56	Ref	0.0014	0.0033	0.65
Cardiovascular and renal characteristics								
Systolic office BP (mm Hg)	123.0	121.5	126.0		124.6	125.4	127.1	
Adjusted median difference	Ref	1.87	5.09	0.022	Ref	1.42	3.28	0.27
Diastolic office BP (mm Hg)	80.4	78.8	81.2		81.3	82.4	81.8	
Adjusted median difference	Ref	0.46	1.25	0.40	Ref	0.37	0.86	0.61
Systolic ABPM (mm Hg)	119.0	120.8	125.3		124.8	121.0	123.5	
Adjusted median difference	Ref	3.88	10.74	0.0004	Ref	-0.092	-0.18	0.94
Diastolic ABPM (mm Hg)	74.6	76.8	79.2		78.7	75.4	78.3	
Adjusted median difference	Ref	2.46	6.82	0.0046	Ref	0.60	1.20	0.49
Pulse wave velocity (m/s)	7.31	7.21	7.71		8.9	9.2	9.7	
Adjusted median difference	Ref	0.14	0.40	0.32	Ref	0.13	0.37	0.55
Serum creatinine (mg/dl)	0.77	0.76	0.78		0.87	0.85	0.88	
Adjusted median difference	Ref	0.0026	0.0070	0.75	Ref	0.0013	0.0031	0.93
Proteinuria (g/24-h)	0.14	0.14	0.15		0.16	0.18	0.13	
Adjusted median difference	Ref	0.0056	0.015	0.35	Ref	-0.012	-0.028	0.33
Albumin/creatinine ratio (mg/g)	1.53	0.79	11.05		32.3	22.2	24.7	
Adjusted median difference	Ref	4.19	11.44	0.0005	Ref	-6.74	-15.4	0.18
eGFR (mL/min/1.73 m^2)	108.9	112.2	108.9		83.4	85.7	83.4	
Adjusted median difference	Ref	0.15	0.40	0.88	Ref	0.22	0.50	0.87

*p-Value for linear trend. Ref: reference value. LDLc low-density lipoproteins cholesterol; HDLc high-density lipoproteins cholesterol; TG/HDLc triglycerides/high-density lipoproteins cholesterol ratio; HOMA-r Homeostasis Model Assessment of Insulin Resistance; 25(OH)D 25-hydroxy-vitamin-D; BMI body mass index; BP blood pressure; ABPM ambulatory blood pressure monitoring (n =193); eGFR estimated glomerular filtration rate, derived

from the Chronic Kidney Disease Epidemiology Collaboration equation. Pulse wave velocity (*n* =174), and albumin/creatinine (*n* =155). Multivariable models were adjusted for continuous variables: BMI, daily energy intake and for categorical variables: sex, smoking status (current smoking: yes/no), physical activity (inactive < 600 mets/min per week, active ≥ 600 mets/min per week) and education (≤ 9 years, > 9 years). For 25(OH)D models were additionally adjusted for season (summer, autumn, winter, spring), and for blood pressure for use of antihypertensive drugs (yes/no).

Table S5. General adjusted Odds Ratio (OR), 95% confidence intervals (CI) for continuous variables according to tertiles (T) of dietary pattern in Uruguayan adults.

Variables	Meat DP					Prudent DP					Cereal and Mate DP				
	T1	T2		T3		T1	T2		T3		T1	T2		T3	
	Ref	OR	95% CI	OR	95% CI	Ref	OR	95% CI	OR	95% CI	Ref	OR	95% CI	OR	95% CI
Systolic blood pressure by office measurement (mm Hg)															
Adjusted	1.00	1.04	0.62-1.75	1.10	0.61-2.00	1.00	0.95	0.57-1.59	1.11	0.67-1.84	1.00	0.91	0.54-1.51	1.06	0.62-1.82
Diastolic blood pressure by office measurement (mm Hg)															
Adjusted	1.00	1.25	0.75-2.11	0.94	0.52-1.70	1.00	0.94	0.55-1.57	1.53	0.92-2.55	1.00	1.09	0.66-1.82	1.13	0.66-1.94
Systolic blood pressure by ABPM measurement (mm Hg)															
Adjusted	1.00	1.58	0.85-2.93	1.64	0.79-3.41	1.00	1.08	0.56-2.08	0.76	0.40-1.43	1.00	0.74	0.39-1.41	0.84	0.44-1.62
Diastolic blood pressure by ABPM measurement (mm Hg)															
Adjusted	1.00	1.14	0.61-2.12	1.51	0.73-3.13	1.00	0.99	0.51-1.90	1.06	0.56-2.01	1.00	0.89	0.47-1.70	1.02	0.53-1.95
Pulse wave velocity (m/s)															
Adjusted	1.00	1.88	0.97-3.66	1.27	0.59-2.73	1.00	2.04	0.98-4.23	1.32	0.67-2.62	1.00	1.01	0.51-1.99	1.26	0.61-2.63
TG/HDLc ratio															
Adjusted	1.00	0.76	0.45-1.27	1.53	0.85-2.78	1.00	1.58	0.94-2.65	1.22	0.73-2.04	1.00	0.71	0.43-1.19	0.81	0.47-1.39
Body mass index (kg/m^2)															
Adjusted	1.00	1.34	0.80-2.26	1.25	0.69-2.26	1.00	2.03	1.21-3.41	1.15	0.69-1.91	1.00	1.11	0.66-1.85	1.23	0.72-2.11
Waist-to-height ratio															
Adjusted	1.00	1.18	0.70-2.00	1.38	0.76-2.51	1.00	0.99	0.59-1.68	0.80	0.48-1.34	1.00	0.71	0.42-1.19	0.99	0.58-1.72
Serum 25(OH)D (ng/ml)															
Adjusted	1.00	0.63	0.37-1.07	1.06	0.58-1.93	1.00	0.90	0.53-1.51	1.13	0.68-1.88	1.00	1.73	1.03-2.91	1.14	0.65-1.99
eGFR (mL/min/1.73m^2)															
Adjusted	1.00	0.86	0.51-1.44	0.48	0.27-0.88	1.00	0.70	0.42-1.17	0.86	0.52-1.43	1.00	1.23	0.74-2.06	1.05	0.61-1.80

Total *n* =294. Ref: reference values. ABPM ambulatory blood pressure monitoring (*n* =193); TG triglycerides; HDLc high-density lipoproteins cholesterol; 25(OH)D 25-Hydroxy-Vitamin-D. eGFR estimated glomerular filtration rate. Pulse wave velocity (*n* =174). Models adjusted for continuous variables: age, body mass index, daily energy intake and categorical variables: sex, smoking status (current smoking: yes/no), physical activity (inactive < 600 mets/min per week, active ≥ 600 mets/min per week) and education (≤ 9 years, > 9 years). For 25(OH)D they were additionally adjusted for season (summer, autumn, winter, spring), and for BP for use of antihypertensive drugs (yes/no).

Table S6. General adjusted Odds ratio (OR) and 95% confidence intervals (CI) for cardiovascular and renal risk phenotypes according to tertiles (T) of dietary pattern and age categories in Uruguayan adults.

Age ≤ 54 y															
**Variables**	**Meat DP**					**Prudent DP**					**Cereal and Mate DP**				
	**T1**	**T2**		**T3**		**T1**	**T2**		**T3**		**T1**	**T2**		**T3**	
	**Ref**	**OR**	**95% CI**	**OR**	**95% CI**	**Ref**	**OR**	**95% CI**	**OR**	**95% CI**	**Ref**	**OR**	**95% CI**	**OR**	**95% CI**
Uncontrolled hypertension by office measurement (SBP ≥140 mm Hg or DBP ≥ 90 mm Hg)															
Adjusted	1.00	2.38	0.30-18.34	0.38	0.031-4.50	1.00	2.70	0.34-21.32	3.59	0.43-30.14	1.00	0.14	0.011-1.84	0.85	0.13-5.65
Uncontrolled hypertension by ABPM measurement (SBP ≥130 mm Hg or DBP ≥ 80 mm Hg)															
Adjusted	1.00	0.36	0.050-2.55	1.68	0.26-10.75	1.00	2.41	0.51-11.48	1.11	0.20-6.19	1.00	6.73	1.04-43.60	2.11	0.34-13.19
High TG/HDLc ratio (>3.75 for men and >3.0 for women)															
Adjusted	1.00	1.38	0.35-5.44	2.90	0.75-11.12	1.00	0.93	0.35-2.49	0.70	0.25-2.00	1.00	0.81	0.29-2.32	0.60	0.19-1.86
Overweight (BMI ≥ 25 < 30 kg/m^2)															
Adjusted	1.00	0.73	0.25-2.20	0.51	0.16-1.59	1.00	0.90	0.34-2.39	1.29	0.51-2.27	1.00	2.87	1.04-7.89	2.36	0.79-7.09
Overweight and Obesity (BMI >25 kg/m^2)															
Adjusted	1.00	2.13	0.63-7.23	1.30	0.39-4.36	1.00	1.51	0.51-4.46	0.71	0.27-1.91	1.00	2.84	0.97-8.28	2.29	0.77-6.87
High waist-to-height ratio (≥0.5)															
Adjusted	1.00	0.38	0.053-2.69	1.30	0.16-10.24	1.00	0.61	0.10-3.71	0.55	0.10-2.89	1.00	0.41	0.074-2.25	2.69	0.32-22.81

Age > 54 y															
**Variables**	**Meat DP**					**Prudent DP**					**Cereal and Mate DP**				
	**T1**	**T2**		**T3**		**T1**	**T2**		**T3**		**T1**	**T2**		**T3**	
	**Ref**	**OR**	**95% CI**	**OR**	**95% CI**	**Ref**	**OR**	**95% CI**	**OR**	**95% CI**	**Ref**	**OR**	**95% CI**	**OR**	**95% CI**
Uncontrolled hypertension by office measurement (SBP ≥140 mm Hg or DBP ≥ 90 mm Hg)															
Adjusted	1.00	0.99	0.44-2.23	0.48	0.15-1.54	1.00	0.96	0.36-2.51	1.21	0.47-3.06	1.00	1.99	0.78-5.09	1.29	0.48-3.49
Uncontrolled hypertension by ABPM measurement (SBP ≥130 mm Hg or DBP ≥ 80 mm Hg)															
Adjusted	1.00	0.95	0.41-2.18	1.83	0.69-4.82	1.00	0.81	0.33-1.97	1.01	0.43-2.35	1.00	1.26	0.53-3.01	0.95	0.40-2.28
Arterial stiffness (pulse wave velocity > 10 m/s)															
Adjusted	1.00	2.86	0.84-9.80	2.14	0.46-10.00	1.00	4.05	0.97-16.93	1.76	0.49-6.34	1.00	0.95	0.28-3.27	0.55	0.14-2.26
High TG/HDLc ratio (>3.75 for men and >3.0 for women)															
Adjusted	1.00	0.94	0.41-2.13	0.44	0.14-1.37	1.00	2.34	0.90-6.12	1.00	0.38-2.64	1.00	0.39	0.15-0.99	0.74	0.29-1.92

Overweight (BMI ≥ 25 < 30 kg/m²)															
Adjusted	1.00	1.71	0.78-3.77	1.25	0.46-3.40	1.00	1.42	0.59-3.46	1.57	0.65-3.78	1.00	1.57	0.64-3.86	2.84	1.11-7.27
Overweight and Obesity (BMI >25 kg/m²)															
Adjusted	1.00	1.56	0.55-4.42	0.59	0.15-2.36	1.00	10.9	2.6-46.2	2.44	0.79-7.51	1.00	1.70	0.57-5.05	2.76	0.80-9.50
High waist-to-height ratio (≥0.5)															
Adjusted	1.00	0.72	0.080-6.45	0.63	0.061-6.58	1.00	5.72	0.13-244.9	0.58	0.079-4.26	1.00	0.45	0.050-4.08	0.64	0.051-8.18
Vitamin D deficiency [serum 25(OH)D <12 ng/ml]															
Adjusted	1.00	2.44	0.54-11.09	1.49	0.11-21.13	1.00	0.20	0.028-1.49	0.063	0.005-0.75	1.00	0.34	0.047-2.43	0.87	0.14-5.29
Chronic kidney disease (eGFR < 60 mL/min/1.73m² or ACR > 30 mg/g)															
Adjusted	1.00	0.64	0.11-3.75	1.31	0.21-8.27	1.00	2.96	0.49-17.68	3.61	0.56-23.48	1.00	3.33	0.54-20.64	2.94	0.40-21.61
Nephrolithiasis (yes)															
Adjusted	1.00	0.63	0.22-1.84	0.94	0.25-3.51	1.00	0.49	0.16-1.54	0.57	0.18-1.82	1.00	1.12	0.37-3.41	0.58	0.17-1.96

Ref: reference values. SBP systolic blood pressure; DBP diastolic blood pressure; ABPM ambulatory blood pressure monitoring (*n* =193); TG triglycerides; HDLc high-density lipoproteins cholesterol; BMI body mass index; 25(OH)D 25-Hydroxy-Vitamin-D. eGFR estimated glomerular filtration rate; ACR: urinary albumin to creatinine ratio.

Arterial stiffness was defined as a pulse wave velocity > 10 m/s (*n* =174). Chronic kidney disease was defined as an eGFR < 60 mL/min/1.73m² or an ACR > 30 mg/g based on a single determination (*n* =155). Nephrolithiasis was defined according to report (yes/no). Multivariable: adjusted for continuous variables: BMI, daily energy intake and categorical variables: sex, smoking status (current smoking: yes/no), physical activity (inactive < 600 mets/min per week, active ≥ 600 mets/min per week) and education (≤ 9 years, > 9 years). For 25(OH)D they were additionally adjusted for season (summer, autumn, winter, spring), and for BP for use of antihypertensive drugs (yes/no). Results for hypertension, and in younger participants (≤ 54 years) additionally arterial stiffness, vitamin D deficiency, chronic kidney disease and nephrolithiasis are not shown as there is possibly a quasi-complete separation of data points and validity of the model fit is questionable.

Table S7. General adjusted Odds ratio (OR) and 95% confidence intervals (CI) for cardiovascular and renal risk phenotypes according to tertiles (T) of dietary pattern and sex in Uruguayan adults

Women															
Variables	Meat DP					Prudent DP					Cereal and Mate DP				
	T1	T2		T3		T1	T2		T3		T1	T2		T3	
	Ref	OR	95% CI	OR	95% CI	Ref	OR	95% CI	OR	95% CI	Ref	OR	95% CI	OR	95% CI
Hypertension (Office SBP ≥140 mm Hg or DBP ≥ 90 mm Hg or use of antihypertensive drugs)															
Adjusted	1.00	2.04	0.56-7.54	0.19	0.016-2.29	1.00	1.03	0.24-4.38	1.25	0.31-4.99	1.00	1.49	0.38-5.89	0.64	0.12-3.50
Uncontrolled hypertension by office measurement (SBP ≥140 mm Hg or DBP ≥ 90 mm Hg)															
Adjusted	1.00	1.23	0.51-2.96	0.38	0.097-1.50	1.00	0.95	0.34-2.66	1.73	0.67-4.49	1.00	1.51	0.58-3.94	1.20	0.40-3.64
Uncontrolled hypertension by ABPM measurement (SBP ≥130 mm Hg or DBP ≥ 80 mm Hg)															
Adjusted	1.00	1.03	0.37-2.86	0.95	0.26-3.52	1.00	1.02	0.33-3.17	1.19	0.40-3.49	1.00	2.42	0.73-8.02	2.78	0.77-9.99
Arterial stiffness (pulse wave velocity > 10 m/s)															
Adjusted	1.00	3.03	0.61-15.0	3.03	0.36-25.5	1.00	15.3	1.87-125.7	2.91	0.56-15.11	1.00	1.10	0.26-4.60	0.64	0.12-3.59
High TG/HDLc ratio (>3.0)															
Adjusted	1.00	1.06	0.48-2.31	1.24	0.47-3.28	1.00	1.08	0.49-2.40	0.63	0.28-1.43	1.00	0.61	0.28-1.34	0.75	0.30-1.85
Overweight (BMI ≥ 25 < 30 kg/m^2)															
Adjusted	1.00	2.04	0.95-4.38	0.91	0.35-2.37	1.00	1.28	0.58-2.80	1.18	0.54-2.57	1.00	1.47	0.67-3.23	1.43	0.58-3.49
Overweight and Obesity (BMI >25 kg/m^2)															
Adjusted	1.00	1.11	0.48-5.40	0.07	0.51-0.02	1.00	3.27	1.21-8.83	1.09	0.47-2.50	1.00	2.33	1.00-5.39	2.54	0.95-6.77
High waist-to-height ratio (≥0.5)															
Adjusted	1.00	0.61	0.16-2.32	1.96	0.43-8.89	1.00	0.89	0.19-4.12	0.70	0.19-2.60	1.00	0.42	0.11-1.63	0.86	0.17-4.34
Vitamin D deficiency [serum 25(OH)D <12 ng/ml]															
Adjusted	1.00	8.53	1.92-37.84	6.03	0.87-41.76	1.00	0.40	0.10-1-53	0.23	0.048-1.09	1.00	0.62	0.17-2.23	0.80	0.19-3.42
Chronic kidney disease (eGFR < 60 mL/min/1.73m^2 or ACR > 30 mg/g)															
Adjusted	1.00	1.94	0.17-22.5	4.75	0.25-90.20	1.00	1.38	0.14-13.48	4.89	0.62-38.68	1.00	2.14	0.16-28.33	4.60	0.34-63.0
Nephrolithiasis (yes)															
Adjusted	1.00	0.88	0.24-3.17	1.45	0.30-6.88	1.00	1.35	0.37-4.97	1.19	0.31-4.56	1.00	1.08	0.34-3.44	0.18	0.027-1.15

Men			
Variables	Meat DP	Prudent DP	Cereal and Mate DP

	T1	T2		T3		T1	T2		T3		T1	T2		T3	
	Ref	OR	95% CI	OR	95% CI	Ref	OR	95% CI	OR	95% CI	Ref	OR	95% CI	OR	95% CI
Hypertension (Office SBP ≥140 mm Hg or DBP ≥ 90 mm Hg or use of antihypertensive drugs)															
Adjusted	1.00	4.73	0.39-58.16	0.81	0.075-8.78	1.00	9.70	0.44-213.2	13.27	0.64-275.8	1.00	0.51	0.047-5.41	0.33	0.031-3.55
Uncontrolled hypertension by office BP measurement (SBP ≥140 mm Hg or DBP ≥ 90 mm Hg)															
Adjusted	1.00	1.31	0.32-5.41	0.68	0.13-3.47	1.00	2.86	0.62-13.28	2.28	0.44-11.89	1.00	0.22	0.034-1.48	0.38	0.089-1.59
Uncontrolled hypertension by ABPM (SBP ≥130 mm Hg or DBP ≥ 80 mm Hg)															
Adjusted	1.00	0.81	0.17-3.84	9.02	1.32-61.5	1.00	0.45	0.093-2.22	0.74	0.15-3.57	1.00	0.55	0.089-3.45	0.19	0.036-1.04
Arterial stiffness (pulse wave velocity > 10 m/s)															
Adjusted	1.00	3.74	0.30-46.62	8.63	0.27-274.4	1.00	0.49	0.028-8.51	0.60	0.041-8.81	1.00	0.14	0.003-6.24	0.08	0.002-3.54
High TG/HDLc ratio (>3.75)															
Adjusted	1.00	0.66	0.16-2.66	0.97	0.25-3.87	1.00	3.17	0.90-11.22	1.65	0.44-6.19	1.00	0.41	0.092-1.85	0.90	0.27-2.94
Overweight (BMI ≥ 25 < 30 kg/m²)															
Adjusted	1.00	0.50	0.15-1.67	0.71	0.21-2.45	1.00	0.87	0.30-2.57	2.66	0.87-8.15	1.00	4.00	1.13-14.17	6.57	1.99-21.67
Overweight and Obesity (BMI >25 kg/m²)															
Adjusted	1.00	0.31	0.050-1.99	0.40	0.060-2.75	1.00	2.70	0.65-11.23	3.11	0.69-14.1	1.00	2.36	0.47-11.7	2.36	0.58-9.73
Nephrolithiasis (yes)															
Adjusted	1.00	0.91	0.20-4.15	1.58	0.31-8.08	1.00	0.19	0.039-0.90	0.36	0.076-1.70	1.00	1.16	0.24-5.53	1.06	0.26-4.38

Ref: reference values. SBP systolic blood pressure; DBP diastolic blood pressure; ABPM ambulatory blood pressure monitoring; TG triglycerides; HDLc high-density lipoproteins cholesterol; BMI body mass index; 25(OH)D 25-Hydroxy-Vitamin-D. eGFR estimated glomerular filtration rate; ACR: urinary albumin to creatinine ratio.

Arterial stiffness was defined as a pulse wave velocity > 10 m/s (*n* =174). Chronic kidney disease was defined as an eGFR < 60 mL/min/1.73m² or an ACR > 30 mg/g based on a single determination (*n* =155). Nephrolithiasis was defined according to report (yes/no). Models adjusted for continuous variables: age, BMI, daily energy intake and categorical variables: smoking status (current smoking: yes/no), physical activity (inactive < 600 mets/min per week, active ≥ 600 mets/min per week) and education (≤ 9 years, > 9 years). For 25(OH)D they were additionally adjusted for season (summer, autumn, winter, spring), and for BP for use of antihypertensive drugs (yes/no). In men, results for waist-to-height ratio, vitamin D deficiency and chronic kidney disease are not shown as there is possibly a quasi-complete separation of data points and validity of the model fit is questionable.
